# Switching Between Bicyclic and Linear Peptides — The Sulfhydryl-Specific Linker TPSMB Enables Reversible Cyclization of Peptides

**DOI:** 10.3389/fchem.2018.00484

**Published:** 2018-10-16

**Authors:** Christoph Ernst, Johannes Heidrich, Catharina Sessler, Julia Sindlinger, Dirk Schwarzer, Pierre Koch, Frank M. Boeckler

**Affiliations:** ^1^Department of Pharmacy and Biochemistry, Institute of Pharmaceutical Sciences, Eberhard Karls Universität Tübingen, Tübingen, Germany; ^2^Department of Pharmacy and Biochemistry, Interfaculty Institute of Biochemistry, Eberhard Karls Universität Tübingen, Tübingen, Germany; ^3^Center for Bioinformatics Tübingen (ZBIT), Eberhard Karls Universität Tübingen, Tübingen, Germany

**Keywords:** reversible peptide cyclisation, sulfhydryl-specific linkers, bicyclic peptides, site-selective disulfide modification, phage display

## Abstract

Phage display-selected bicyclic peptides have already shown their great potential for the development as bioactive modulators of therapeutic targets. They can provide enhanced proteolytic stability and improved membrane permeability. Molecular design of new linker molecules has led to a variety of new synthetic approaches for the generation of chemically constrained cyclic peptides. This diversity can be useful for the development of novel peptide-based therapeutic, diagnostic, and scientific tools. Herein, we introduce 1,3,5-tris((pyridin-2-yldisulfanyl)methyl)benzene (TPSMB) as a planar, trivalent, sulfhydryl-specific linker that facilitates reversible cyclization and linearization via disulfide bond formation and cleavage of bicyclic peptides of the format CX_n_CX_n_C, where X is any proteinogenic amino acid except cysteine. The rapid and highly sulfhydryl-specific reaction of TPSMB under physiological conditions is demonstrated by selecting bicyclic peptide binders against c-Jun N-terminal kinase 3 (JNK3) as a model target. While model peptides remain stably cyclized for several hours in presence of typical blood levels of glutathione *in vitro*, high cytosolic concentrations of glutathione linearize these peptides completely within 1 h. We propose that reversible linkers can be useful tools for several technical applications where target affinity depends on the bicyclic structure of the peptide.

## Introduction

**Figure 1:**
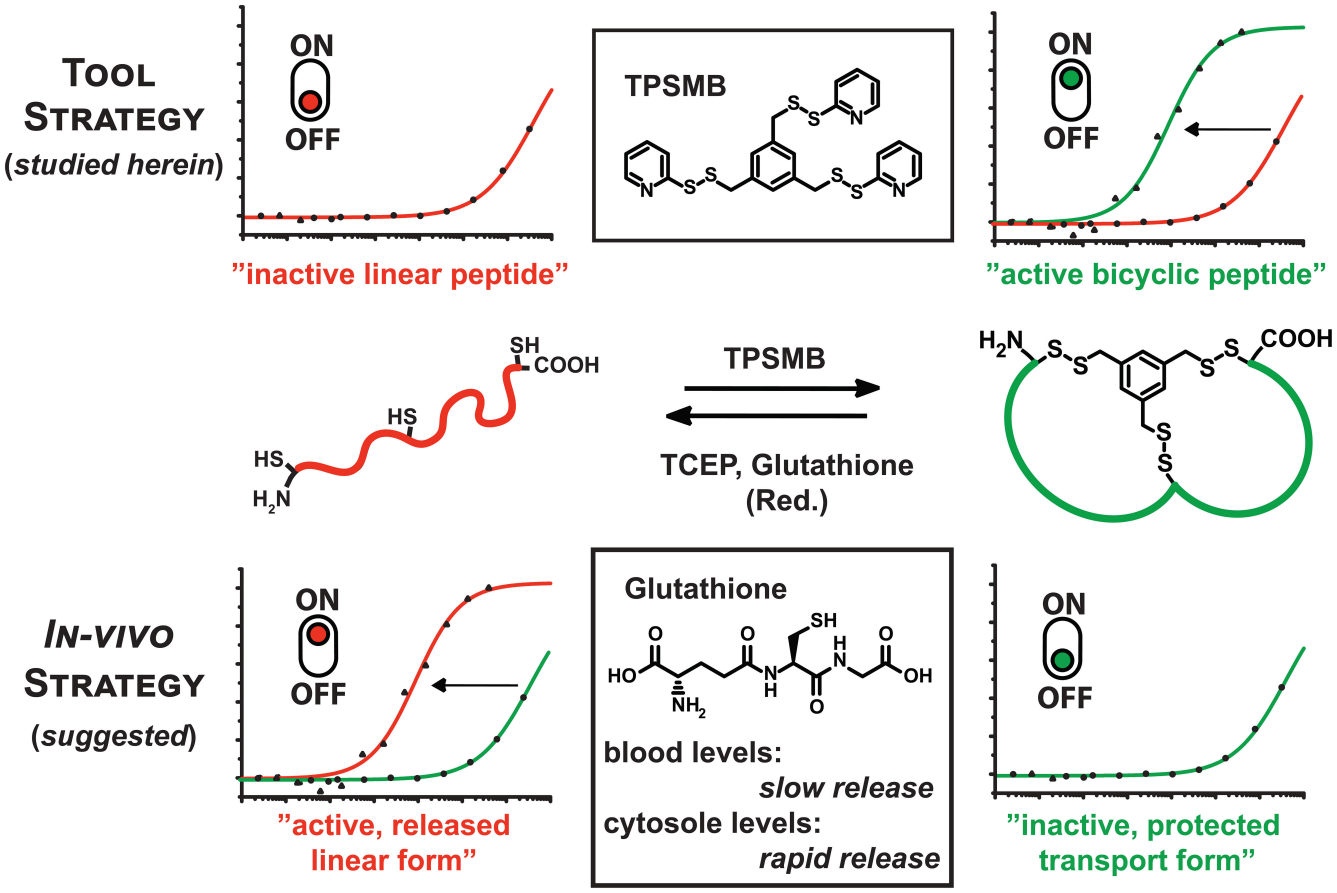
Graphical representation of the reaction of TPSMB with a peptide of the format CX_n_CX_n_C. The resulting disulfide-based modification can be selectively reduced by mild reducing agents like glutathione, β-mercaptoethanol or TCEP. Based on the additive *in vitro* or the cellular environment the equilibrium can be switched from linear to cyclized and vice versa determining the target affinity or bioactivity of the peptide.

Short peptides binding therapeutic targets with high affinity have been suggested to unite the advantages of the enhanced binding diversity of biologicals and the small molecules capability to cross the cell membrane (Bruno et al., 2013; Loktev et al., 2017). However, the applicability of peptides as drugs is still limited by their proteolytic instability and limited membrane permeability (Qian et al., 2015). To overcome this issue, chemical peptide cyclisation (Nguyen et al., 2010) or stapling (Walensky and Bird, 2014) strategies have been employed to improve the proteolytic stability. Enhanced membrane permeability of peptides could be observed by structurally rigidified arginine-rich peptides (Lättig-Tünnemann et al., 2011). Furthermore, it was recently reported by Pei and coworkers that cyclic peptides containing amphipathic sequences (e.g., FΦRRRR, Φ = L-2-naphthylalanine) efficiently enter mammalian cells through endocytosis (Qian et al., 2013, 2014; Oh et al., 2014). By incorporating these short sequence motifs into the bicyclic peptide, this approach facilitates the delivery of biologically active cyclic peptides into the cytosol and nucleus of mammalian cells (Qian et al., 2013, 2014; Lian et al., 2014). Selective chemical modification strategies are able to extend the scope of applications not only in a therapeutic manner, but also for the development of novel tools for protein capturing, bioimaging and targeted drug delivery (Bruno et al., 2013). Molecular design of new linker scaffolds can further broaden the spectrum of structured cyclic peptides (Chen et al., 2012; Ernst et al., 2018). Herein, we present the synthesis of the novel trivalent, sulfhydryl-specific linker 1,3,5-tris((pyridin-2-yldisulfanyl)methyl)benzene (**4**, TPSMB), that enables the generation of bicyclic peptides modified via three pyridyl-activated disulfide groups (see Figure 1).

The resulting disulfide-based modification can be selectively reduced by mild reducing agents like glutathione, β-mercaptoethanol, or tris(2-carboxyethyl)phosphine (TCEP). This gives access to two new strategies: Firstly, the rapid and highly sulfhydryl-specific reaction under physiological conditions enables the implementation of this linker in phage display-based selection of bicyclic peptides. Assuming that the reduction of the disulfide bond and cleavage of the linker significantly weakens the affinity toward the target, the novel TPSMB linker provides the possibility to switch between the “active” and “inactive” peptide form. Immobilized peptide binders on surfaces or beads could reversibly capture and release target proteins, controlled by mild reducing agents. Secondly, the reversible peptide modification delivers a general strategy for delivering linear peptides into mammalian cells (Qian et al., 2015). A number of molecular targets like e.g., PDZ (Doyle et al., 1996; Grootjans et al., 1997) and BIR domains (Wu et al., 2000) require that the peptidyl ligand is in a linear conformation while cyclization or stapling is disrupting the desired target binding. Herein, we report, based on the idea of Pei and coworkers (Qian et al., 2015), another potential strategy for delivering linear peptides into mammalian cells through reversible disulfide-mediated cyclisation. In the oxidizing extracellular or endosomal milieu, the peptide exists as cyclic and rigidified peptide with enhanced proteolytic stability and cell permeability. Once the bicyclic peptide has entered the intracellular space the disulfide-based modification is reduced by high intracellular levels of glutathione, releasing the linear, biologically active peptide (Jha et al., 2011). Reduced glutathione (GSH) occurs in the cytosol of cells in a concentration range of 1–10 mM (Meister, 1988), whereas the GSH concentration in the majority of tissues is about 1–2 mM. Only in hepatocytes the GSH concentration can reach about 10 mM (Forman et al., 2009). The plasma concentration of GSH is in the micromolar range 3–4 μM (Michelet et al., 1995). With the advent of phage display and other *in vivo* evolution techniques it has become possible to select cyclic peptide binders against many diverse target proteins. A robust and widely applied approach is based on the cyclization of peptides displayed on phage via a disulfide bridge. A relatively new cyclic peptide format developed using phage display involves bicyclic peptides (Lian et al., 2014; Deyle et al., 2017; Zorzi et al., 2017). These molecules consist of two macrocyclic peptide rings cyclized through a chemical multivalent linker molecule. Compared to monocyclic peptides of comparable molecular mass, bicyclic peptides are more constrained in their conformation. As a result, they can bind to their targets with a higher affinity and are more resistant to proteolytic degradation. Phage-encoded bicyclic peptides are generated by chemically cyclizing random peptide libraries on phage. Binders are identified by conventional phage panning and DNA sequencing (Deyle et al., 2017; Zorzi et al., 2017).

## Results and discussion

### Chemistry

**Figure 2:**
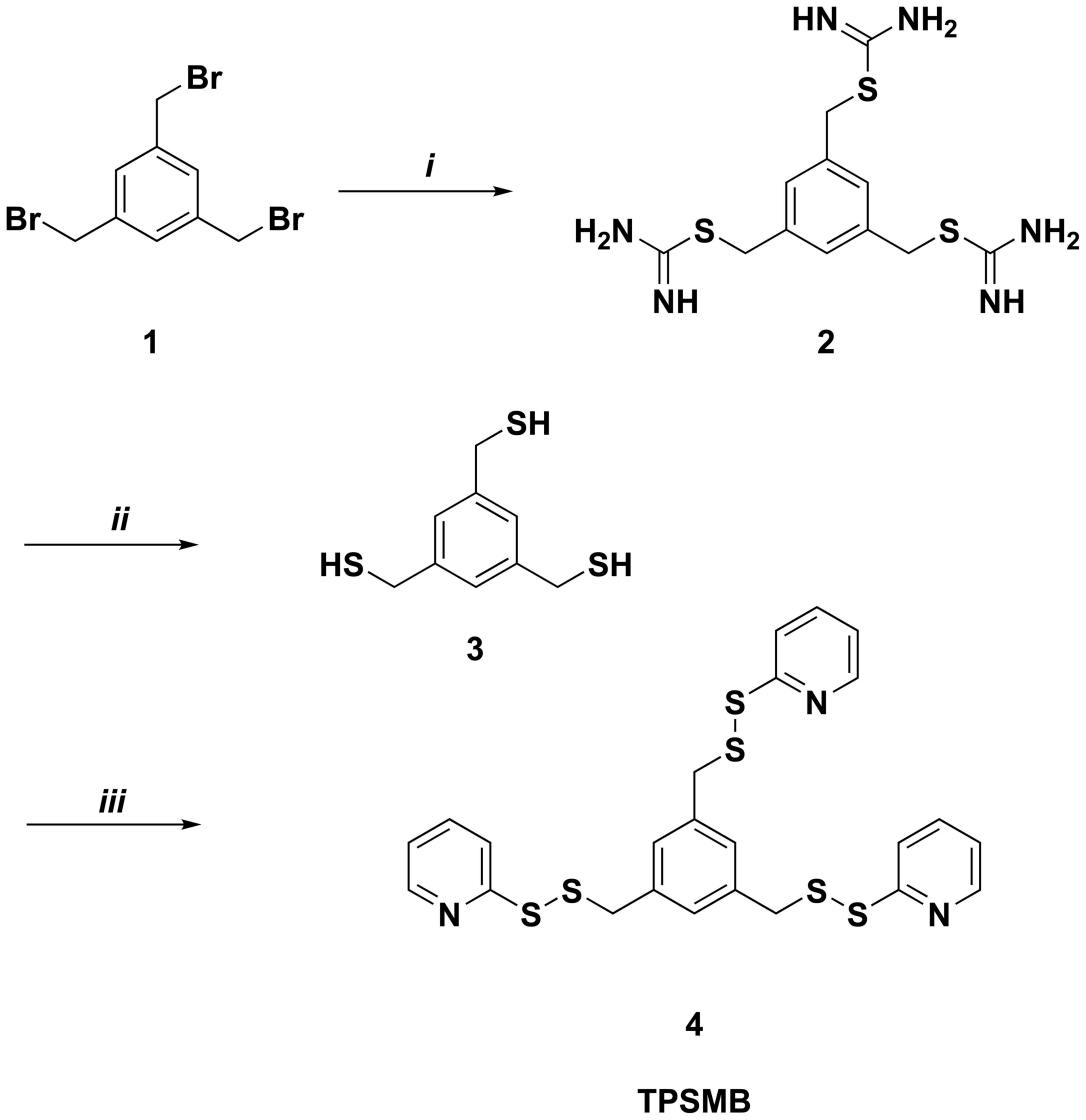
Synthesis of 1,3,5-tris((pyridin-2-yldisulfanyl)methyl)benzene (TPSMB). Reagents and conditions: (*i*) thiourea, acetone, reflux, 2 h (*ii*) NaOH, reflux, 2 h (*iii*) benzotriazole, 1-chlorobenzotriazole, 2-mercaptopyridine, dichloromethane, −78 to 0°C, 1 h.

Benzene-1,3,5-triyltrimethanethiol (**3**) was generated by refluxing 1,3,5-tris(bromomethyl)benzene (**1**) with thiorurea in acetone for 2 h (Figure 2). The resulting benzene-1,3,5-triyltris(methylene)tricarbamimidothioate (**2**) was obtained by filtration and subsequently hydrolyzed under reflux conditions for 2 h in aqueous sodium hydroxide. After purification by column chromatography, benzene-1,3,5-triyltrimethanethiol (**3**) was obtained with a yield of 91%. The activated unsymmetrical disulfide bond of the desired TPSMB linker **4** was synthesized as following. First, 2-mercaptopyridine was converted at −78°C in dichloromethane with benzotriazole and 1-chlorobenzotriazole into the sulfhydryl reactive 1-(pyridin-2-ylthio)-1*H*-benzo[*d*][1,2,3]triazole intermediate over a time of 30 min. The addition of benzene-1,3,5-triyltrimethanethiol (**3**) at 0°C gave within 30 min the desired 1,3,5-tris((pyridin-2-yldisulfanyl)methyl)benzene (**4**, see Figure 2). After purification over silica, **4** was obtained with a yield of 55% and an overall yield of 50%.

### Characterization of the reversible peptide modification

The application of TPSMB as a linker in phage display-based screening of bicyclic peptides (Heinis et al., 2009; Heinis, 2011) requires a selective and nearly quantitative modification of the peptide library. For the *in vitro* characterization of the sulfhydryl-reactivity of the newly synthesized linker TPSMB, we chose a MALDI-TOF controlled strategy for the optimization of the reaction conditions. As a positive control, we monitored the reaction of the cyclization linker TBMB with the test peptide TPB (NH_2_-ACEGMINSCEKSDYECG-CONH_2_, 1839.1 Da) under reaction conditions published by Heinis et al. (20 mM (NH_4_)_2_CO_3_, 5 mM EDTA, pH 8, 30°C) (Rentero Rebollo and Heinis, 2013) over time by MALDI-TOF mass spectrometry.

**Figure 3:**
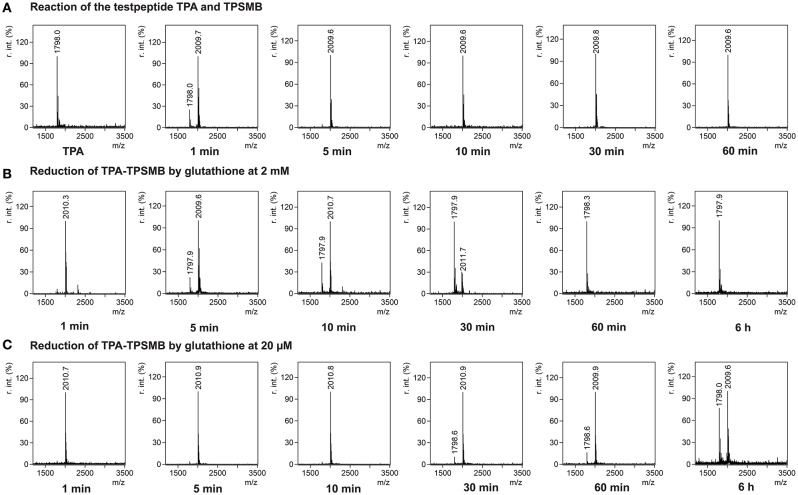
Characterization of the reversible cyclization of test peptide TPA and the designed linker TPSMB. **(A)** Reaction of TPSMB with test peptide TPA (NH_2_-A*C*KMREEV*C*LGPESS*C*G-CONH_2_, 1798.1 Da) monitored by MALDI-TOF over a time of 60 min. Reaction conditions: 20 mM HEPES, 15% acetonitrile, pH 7.0, 30°C, 0.5 mM TPA, 1.2 equivalents TPSMB. **(B)** Reduction of the purified TPSMB-TPA bicyclic peptide with glutathione. Reaction conditions: 2 mM glutathione, PBS, pH 7.4, 30°C, 50 μM TPSMB-TPA. **(C)** Reduction of the purified TPSMB-TPA bicyclic peptide with glutathione. Reaction conditions: 20 μM glutathione, PBS, pH 7.4, 30°C, 50 μM TPSMB-TPA. *Annotation:* Crosslinked, dimerized/trimerized etc. peptides using this protocol were not observed.

In <5 min the linear test peptide TPB was almost quantitatively (>95%) cyclized, forming the desired bicycle peptide TPB-TBMB. However, in the ongoing reaction process (>10 min) the TPB-TBMB bicyclic peptide was also considerably modified by the applied excess (1.5 equivalents) of TBMB (Figure S2). The reaction of TPSMB was investigated under varying reaction conditions. Different buffers, pH-values (6–8), acetonitrile concentrations, and temperatures were tested. The best results were achieved using a HEPES buffer at pH 7, 15% acetonitrile and 30°C (see Figure 3A). Under these conditions the highly selective and fast reaction of pyridyl-activated disulfides gave in <5 min the desired bicyclic TPA-TPSMB product (TPA = NH_2_-ACKMREEVCLGPESSCG-CONH_2_, 1798.1 Da) with no detectable formation of side products so far. Similar results were observed for the reaction of test peptide TPB with TPSMB (Figure S3). Based on these data, we judged that reaction kinetics and selectivity profile of TPSMB are suitable for using TPSMB for peptide selection by phage display. Furthermore, we investigated the reduction of the resulting bicyclic peptide with GSH. The chosen GSH concentrations 20 μM and 2 mM are in accordance with the concentrations found in human plasma (3–4 μM; Michelet et al., 1995) and in the cytosol (1–10 mM; Meister, 1988). After 60 min under the reductive conditions of the cytoplasm (2 mM GSH), a complete reduction of the TPA-TPSMB bicycle could be detected *in vitro* (Figure 3B). In contrast, under plasma conditions (20 μM GSH) only 15% of the peptide was reduced after 1 h (Figure 3C). While measurements after 6 h of reaction showed for 20 μM GSH only a reduction of ~40%, an influence of oxidative effects by oxygenation during 6 h of shaking cannot be excluded. Furthermore, we investigated the reduction of a TPSMB modified peptide with a 10-fold excess of TCEP. Under these conditions the TPSMB bicycle was rapidly and quantitatively reduced in < 15 min (see Figure S3). These results emphasize that the cyclization can be “switched on and off” under suitable reaction conditions *in vitro* within 5–15 min.

### Phage affinity selection of TPSMB-modified peptides

**Figure 4:**
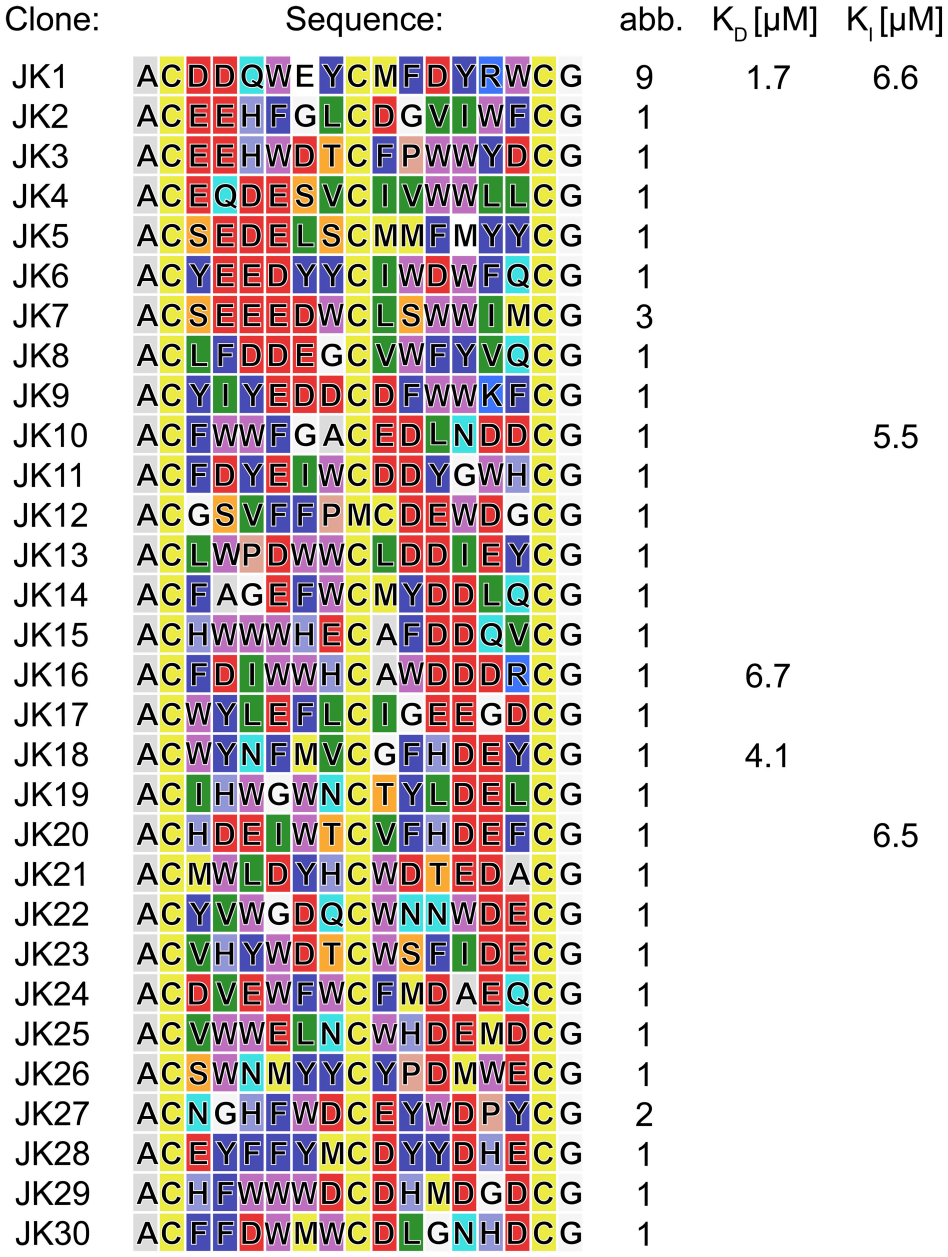
Peptides isolated in phage selections against JNK3. Amino acid sequences are colored in the Rasmol color code. The frequency each peptide sequence was found is indicated. Affinities were determined by FP assays. For complete FP data obtained in direct titration and competitive FP assays (see Tables S2,S3, Figures S4,S5).

The phage display-based methodology for the selection of chemically modified peptides developed by Heinis and Winter (Heinis et al., 2009) served as a framework for the implementation of the newly developed TPSMB linker concept. The phage library with a size of 8.8·10^7^ different clones presenting linear peptides of the format ACX_6_CX_6_CG-phage, where X is any proteinogenic amino acid except cysteine, was prepared with minor modifications as described in the literature (see Supplementary Material; Rentero Rebollo and Heinis, 2013). In general, it has to be assumed that after preparation of the phages, the thiol groups of the cysteine-rich peptide library are partly oxidized. Therefore, it is essential to reduce the potentially oxidized peptides to enable to full reactivity of the sulfhydryl-based modification. Heinis et al. have implemented this reduction of phages via TCEP into their protocol (Heinis et al., 2009; Heinis, 2011; Rentero Rebollo and Heinis, 2013). This includes a stepwise dilution procedure of the reducing agent by using centrifugal filters. At first, selections applying this procedure failed. Because of the sensitivity of the disulfide bonds resulting from TPSMB modification to TCEP, it was required to develop a new purification step that is able to remove even traces of TCEP. Hence, we developed a faster and more effective TCEP removal step by purifying the reduced phages over a Sephadex desalting column. Subsequently, the linear peptides were selectively modified by TPSMB under optimized conditions. The phage-encoded bicyclic peptide library was subjected to three iterative rounds of affinity selection against c-Jun N-terminal kinase 3 (JNK3) using this modification protocol. JNK3 was pragmatically chosen as a model target based on a variety of active and successful drug discovery projects (Goettert et al., 2010; Lange et al., 2015; Muth et al., 2015, 2017; Ansideri et al., 2018) and assay developments (Ansideri et al., 2016, 2017) reported recently by the involved laboratories. After each selection, the captured phages were eluted by treatment with low pH buffer. After the third iterative round, individual clones were selected and sequenced revealing the respective amino acid sequences (Figure 4). The obtained sequences show a predominantly acidic character. More than 50% of the isolated sequences present an acidic pattern of two or even more consecutive acidic amino acids (mostly AspGlu, AspAsp, or GluGlu). Interestingly this pattern could be found at nearly every position within the two peptide loops (Figure 4). In addition to the acidic motif, an apolar motif consisting of two or three consecutive tryptophan, phenylalanine or tyrosine residues was found to appear in most of the sequences in the opposite loop of the acidic motif.

To verify the affinity of the discovered peptides to JNK3, some diverse sequences were chosen containing the acidic motif at different positions of both loops. For the assessment of target binding, fluorescence polarization assays with direct and competitive titration regimes were applied (Ansideri et al., 2016). For the competitive fluorescence polarizations assays, the previously established, fluorescein-labeled fluorescence polarization (FP) probe PIT0105006 was applied, binding with a K_D_-value of 3.0 ± 0.2 nM to JNK3 (Ansideri et al., 2017). All of the selected, TPSMB modified sequences exhibited affinities to JNK3 in the one-digit micromolar range. Sequence JK1 that was identically found with an abundance of nine (~20% of the shown sequences) was analyzed as TPSMB modified bicyclic peptide both in direct and competitive FP experiments. In the direct binding experiments, JK1 revealed the highest affinity with a K_D_-value of 1.7 ± 0.8 μM (direct titration). The slightly weaker K_I_-value of 6.6 μM for JK1-TPSMB determined by competitive FP assay, indicates that this bicyclic peptide occupies at least partly the classical ATP-binding site, thus, competing with the fluorescein-labeled compound PIT0105006.

**Figure 5:**
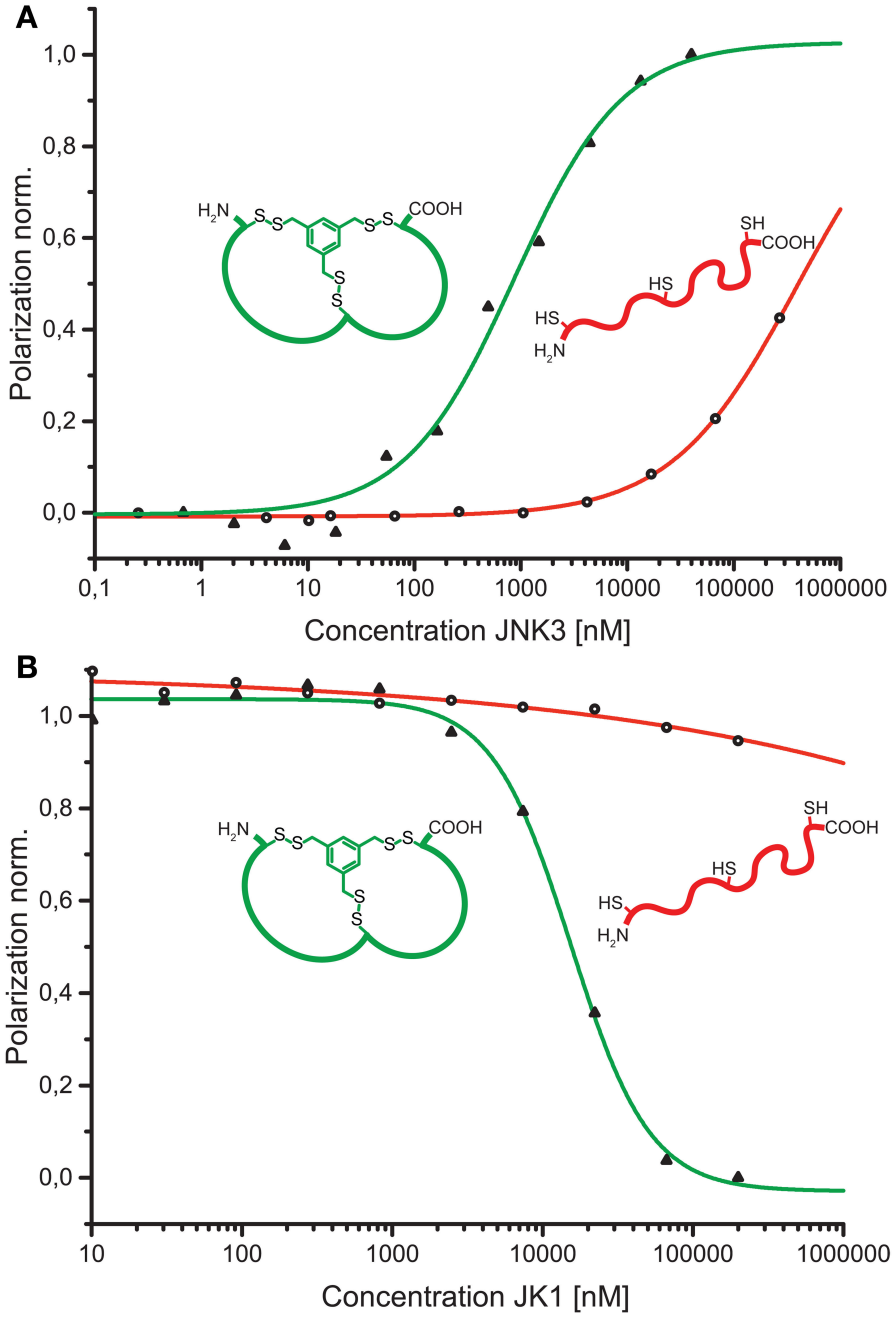
**(A)** Comparison of binding curves obtained from direct binding of fluorescein-labeled bicyclic peptide JK1F-TPSMB (green) or linear peptide JK1F (red) to JNK3. JK1F-TPSMB: K_D_ = 2.6 μM (SE: 0.1 μM); JK1F: K_D_ ≈ 390 μM (SE: 250 μM). **(B)** Comparison of competitive fluorescence polarization assays of unlabeled peptides JK1-TPSMB (green) or JK1 (red) displacing PIT0105006 (K_D_ = 3.0 nM) from binding to JNK3. JK1-TPSMB: IC_50_ = 15.6 μM (SE: 2.6 μM), K_I_ = 3.6 μM; JK1: IC_50_ ≫ 1.000 μM, K_I_ ≫ 230 μM, SE = standard error.

As a conceptual framework of this study, we have suggested to be able to select bicyclic peptides that upon linearization will lose their target affinity. Thus, binding to the target can be switched on and off by the discussed reaction conditions for efficient cyclization or linearization. To showcase this concept, we have analyzed the affinities of the unmodified, linear JK1 peptide in direct and competitive FP measurements. Without cyclisation by TPSMB the affinity toward JNK3 is almost completely lost (K_D_ ≈ 390 μM; K_I_ ≫ 230 μM, see Figure 5).

## Conclusion

Herein, we introduced the new trivalent, sulfhydryl-specific linker TPSMB (**4**), which allows to switch effectively between a bioactive/high affinity state upon cyclization of properly selected peptides and an inactive/low affinity state, when linearizing the peptides due to linker cleavage. This strategy is contrary to the use of classical linkers, such as TBMB, which aim at a persistent modification of the peptide to form a bicyclic structure. We have established efficient reaction conditions for the formation of bicyclic peptides by addition of TPSMB. The resulting bicyclic peptide is modified via three disulfides bonds which can be reduced by rather mild, biocompatible reducing agents, such as e.g., glutathione or TCEP. This reversible peptide modification provides a general strategy for delivering linear peptides into mammalian cells. For this purpose, we investigated *in vitro* the reductive stability of a TPSMB modified peptide against the glutathione concentrations found in the human plasma and in the cytosol. Under the conditions of the cytosol, the linear peptide was released completely after a time of ~60 min. In contrast, under the conditions of plasma glutathione levels, 85% of the peptide still existed in the bicyclic form after 1 h, while even after 6 h more than 50% of the bicyclic structure remained intact. Thus, the TPSMB linker strategy may provide bicycles with enhanced proteolytic stability and cell permeability in the blood that are quickly released into the linear peptides after uptake into the cytosol. However, a possible cytotoxicity of released benzene-1,3,5-triyltrimethanethiol has not yet been investigated. Furthermore, we could show the general applicability of the linker TPSMB for the phage display-based selection of bicyclic peptides modified via disulfide bonds. We were able to exemplify this by selecting and isolating bicyclic peptides that bind recombinant human JNK3 as a model target. FP measurements confirm that all of the six analyzed TPSMB-modified peptides show affinity against the target. Peptide JK1-TPSMB shows in the direct FP experiment the highest affinity with 1.7 ± 0.8 μM. Similar binding of JK1-TPSMB to JNK3 under competitive FP conditions indicates that the bicyclic peptide binds at least partially to the ATP-binding site, displacing the used fluorescein-labeled compound PIT0105006. In contrast, the linear peptide JK1 shows no significant affinity to JNK3 (K_I_ ≫ 230 μM) emphasizing that the affinity can be switched on and off by cyclization and linearization of the peptide *in vitro*. In general, if the affinities of linear and cyclized peptides are found to be too similar, negative selections of the linear peptides can be performed as follow: After each panning round, the eluted phages that bear cyclized peptides can be reduced with TCEP under the same conditions as described for the phage modification step (see Supplementary Material). After reduction, phages bearing peptides that also bind efficiently to the target in their linearized form can be removed by subjecting the reduced, positively selected phage fraction to beads presenting the respective target. All phages not binding to the magnetic beads in this negative selection step are collected and used for the next positive selection. This approach enables the enrichment of peptides that bind the target only in its cyclized form. Applications of this strategy can include, but are not limited to capturing/releasing the target on beads, columns or surfaces. Hence, we suggest that this study is a promising starting point for further developments.

## Experimental section

### Chemicals and reagents

All chemicals and reagents were obtained from commercial sources and used as received, unless noted otherwise. Liquid chromatography-mass spectrometry (LC-MS) was performed on a LC-MS 2020 system from Shimadzu equipped with a Kinetex C18 column (100 × 2.1 mm, 2.6 μm, 100 Å, Phenomenex). All solvents for LC-MS were acquired from Th. Geyer GmbH. Samples were prepared with solvent LCMS-A (0.1% formic acid (FA) in water) and LCMS-B (80% ACN, 0.1% FA in water). The flow rate was 0.2 mL/min with a gradient from 5 to 95% LCMS-B within 12.75 min.

### Benzene-1,3,5-triyltrimethanethiol (3)

A mixture of 1,3,5-tris(bromomethyl)benzene (2.0 g, 5.60 mmol) and thiourea (2.56 g, 33.63 mmol) dissolved in acetone (50 mL) was refluxed for 2 h. The resulting suspension was cooled to 4°C, filtrated and washed with cold acetone (10 mL). The solid isothiouronium salt **2** was resuspended in aqueous sodium hydroxide solution (50 mL, 2 M NaOH) and refluxed for 2 h. Subsequently, the reaction mixture was allowed to cool to room temperature and water (100 mL) was added. First, the aqueous solution was extracted with ethyl acetate (3 × 50 mL). Then, the solution was acidified with diluted hydrochloric acid solution to pH 2 and extracted again with ethyl acetate (3 × 50 mL). The combined organic phases were dried over magnesium sulfate and evaporated under reduced pressure. The resulting yellowish oil was purified by silica column chromatography (eluent: hexane: ethyl acetate = 7: 3) yielding **3**. Yield: 1.1 g (91%). ^1^H-NMR (300 MHz, CDCl_3_) δ [ppm]: 1.79 (s, SH, 3H), 3.20 (s, CH_2_, 6H), 7.18 (s, H_arom_, 3H). ^13^C-NMR (75 MHz, CDCl_3_) δ [ppm]: 28.7, 126.5, 142.1. LC-MS (m/z): calculated 217.38 [M+H^+^]^+^, found 217.5 [M+H^+^]^+^, 215.3 [M-H^+^]^−^, R_f_ (70% hexane : 30% ethyl acetate): 0.72.

### 1,3,5-tris((pyridin-2-yldisulfanyl)methyl)benzene (4)

To a solution of benzotriazole (516.98 mg, 4.34 mmol) and 1-chlorobenzotriazole (1.0 g, 6.51 mmol) in dichloromethane (30 mL, extra dry) we slowly added a solution of 2-mercaptopyridine (482.56 mg, 4.34 mmol) in dichloromethane (5 mL, extra dry) at −78°C. (Hunter et al., 2006) After 30 min, the reaction was allowed to warm to −20°C and benzene-1,3,5-triyltrimethanethiol (**3**) (300 mg, 1.39 mmol) was added in one portion. Then the reaction mixture was allowed to warm to 0°C over a period of 30 min. After this time the reaction was quenched by the addition of aqueous sodium thiosulfate/sodium bicarbonate solution. The resulting crude product was purified by silica column chromatography (eluent: hexane: ethyl acetate = 1: 1) yielding **4**. Yield: 415 mg (55%). ^1^H-NMR (300 MHz, CDCl_3_) δ [ppm]: 3.80 (s, CH_2_, 6H), 6.97 (ddd, 5-CH_pyridine_, 3H, ^3^*J* = 7.3 Hz, ^3^*J* = 4.9 Hz, ^4^*J* = 1.1 Hz), 7.02 (s, H_arom_ 3H), 7.39 (dt, 3-CH_pyridine_, 3H, ^3^*J* = 8.1 Hz, ^4^*J* = 1.1 Hz, ^5^*J* = 1.1 Hz), 7.48 (ddd, 4-CH_pyridine_, 3H, ^3^*J* = 8.1 Hz, ^3^*J* = 7.3 Hz, ^4^*J* = 1.8 Hz), 8.36 (ddd, 6-CH_pyridine_, 3H, ^3^*J* = 4.9 Hz, ^4^*J* = 1.8 Hz, ^5^*J* = 1.1 Hz). ^13^C-NMR (75 MHz, CDCl_3_) δ [ppm]: 43.2, 119.6, 120.7, 129.42, 137.0, 137.4, 149.5, 159.8. LC-MS (m/z): calculated 544.8 [M+H^+^]^+^, found 544.9 [M+H^+^]^+^.

### Peptide cyclization and mass spectrometric analysis

Cyclization of TPA with TPSMB: Test peptides were dissolved in the respective buffer system and placed in a thermoshaker (Bioer Technology) at 30°C. After 10 min of incubation, the respective amount (1.5 equiv.) of linker dissolved in a volume of 100 μL acetonitrile was added to 500 μL test peptide solution (0.5 mM) under shaking. The progress of the reaction was determined by MALDI-TOF mass spectrometry at different times (1, 5, 10, 30, 60 min). Taken samples were directly mixed with the same volume of matrix solution [20 mg α-cyano-4-hydroxycinnamic acid (α-CHCA) in 1 mL 50:50 H_2_O/ACN with 0.1% TFA trifloroacetic acid (final conc.)], spotted on target plate and measured by MALDI-TOF mass spectrometry (Bruker Daltonics-autoflex II, Massachusetts, USA). Reduction of TPA-TPSMB with glutathione: Purified TPA-TPSMB (50 μM) was dissolved in 9.5 mL PBS (pH 7.4) and placed in an incubator with 100 rpm shaking at 30°C. After 10 min of incubation, 0.5 mL of the respective 20-fold concentrated amount of glutathione was added. Reduction of TPA-TPSMB with TCEP: Purified TPA-TPSMB (0.5 mM) was dissolved in 2 mL 20 mM HEPES, 15% ACN, pH 7 and 5 mM TCEP and placed in a heat block at 30°C. The progress of the reduction was determined by MALDI-TOF mass spectrometry at different times (1, 5, 10, 30, 60 min, and 6 h) as described above.

### Phage selection of TPSMB modified peptides

Phage display was performed with modifications as described in the literature (Rentero Rebollo and Heinis, 2013). *E. coli* TG1 glycerol stock cells containing the phage library were used to inoculate 500 mL of 2xYT media containing 30 mg/mL until an OD_600_ of 0.1 was reached. After incubation for 16 h at 30°C the *E. coli* TG1 cells were removed from the phage containing supernatant by centrifugation for 30 min at 6,000 rpm and 4°C. The phages were precipitated by addition of 20% precipitation buffer (PEG buffer, 20% PEG-6000, 2.5 M NaCl). After addition of the PEG buffer the phage suspension was cooled on ice for 60 min and subsequently centrifuged for 30 min at 7,000 rpm and 4°C. After careful removal of the supernatant the resulting phage pellets were resuspended in 10 mL ice-cold buffer R (20 mM HEPES, 5 mM EDTA, pH 8) and again centrifuged for 30 min at 4,000 rpm and 4°C. The phage containing supernatant was carefully transferred into a new 50 mL falcon tube. To the clear phage solution TCEP was added with a final concentration of 1 mM and the reduction was incubated at 42°C for 1 h. In order to remove TCEP the reduced phage solution was purified twice over a desalting column (HiPrep 26/10 Desalting, GE Healthcare). The resulting phage containing fraction was adjusted to 32 mL with buffer R and 4 ml of 100 mM TPSMB solution in acetonitrile was added to obtain a final linker concentration of 11.11 μM and incubated at 30°C for 10 min under gentle shaking. The chemically modified phages were subsequently precipitated by the addition of 0.2% of buffer PEG, cooled down on ice for 30 min and centrifuged at 4,700 rpm for 30 min at 4°C. The phage pellet was dissolved in 3 ml buffer W1 (10 mM Tris-HCl, 150 mM NaCl, 10 mM MgCl_2_, 1 mM CaCl_2_, pH 7.4). Biotinylated JNK3 was immobilized on magnetic streptavidin beads (Dynabeads™ M-280 Streptavidin, Invitrogen) following the protocol provided in Supplementary Material. Negative control without antigen was prepared by addition of buffer W1 instead of antigen. Subsequently, the magnetic beads were washed three times with 0.5 mL buffer W1 and incubated for 30 min at room temperature in 300 μL buffer W1 supplemented with 150 μL blocking buffer W2 (Buffer W1 + 3% BSA, 0.3% Tween 50). At the same time, the chemically modified phages, stored in 3 mL buffer W1, were blocked at room temperature by addition of 1.5 ml of buffer W2 for 30 min. The blocked bead suspension (0.45 ml) and 2.25 mL of the blocked phage suspension were mixed together and incubated for 30 min on a rotating wheel at room temperature. The same was performed for the negative control. The beads were washed eight times with buffer W3 (Buffer W1 + 0.1% Tween 50) and twice with buffer W1. The phages were eluted by incubation with 100 μL of buffer E (50 mM glycine, pH 2) for exactly 5 min and then directly transferred into 50 μL of buffer N (1 M Tris, pH 8) for neutralization. The eluted phages were added to 25 mL of *E. coli* TG1 cells at OD_600_ of 0.4 for 90 min at 37°C. After centrifugation for 5 min at 4,000 rpm and 4°C the cell pellets of positive and negative experiments were plated on each two large 2xYT/chloramphenicol (30 mg/mL) plates. For each round the input and output phage titer was determined. Second and third rounds of panning were performed following the same procedure but using in the second round instead of streptavidin beads neutravidin coated magnetic beads. Magnetic neutravidin beads were prepared by reacting 1 mg neutravidin (Pierce) with 0.5 mL tosyl-activated magnetic beads (Dynabeads™ M-280 Tosylactivated, Invitrogen) according to the supplier's instructions. After round two and three individual clones were picked, amplified and extracted plasmid DNA sequenced using primer *seqba* (Eurofins, Germany).

## Author contributions

**4** (TPSMB) was conceived of and designed by CE and FB. Synthesis was planned by CE, PK, and FB. Peptide synthesis, purification, and characterization was performed by CE and supported by JS and DS. JH expressed and purified JNK3. Fluorescence polarization assays with JNK3 were done by CS. CE performed all other chemical, analytical, and biological experiments. CE, FB, PK, and DS analyzed data and prepared the manuscript.

### Conflict of interest statement

The authors declare that the research was conducted in the absence of any commercial or financial relationships that could be construed as a potential conflict of interest.
