# Supplementary material for: Switching Between Bicyclic and Linear Peptides — The Sulfhydryl-Specific Linker TPSMB Enables Reversible Cyclization of Peptides
Source: Front Chem. 2018 Oct 16;6:484. doi: 10.3389/fchem.2018.00484 (PMC6198510; doi:10.3389/fchem.2018.00484)

Supplementary Material

Switching between Bicyclic and Linear Peptides – The Sulfhydryl-specific Linker TPSMB Enables Reversible Cyclization of Peptides

Christoph Ernst, Johannes Heidrich, Catharina Sessler, Julia Sindlinger, Dirk Schwarzer, Pierre Koch, and Frank M. Boeckler*

*** Correspondence:** Frank M. Boeckler: frank.boeckler@uni-tuebingen.de

I: **Preparation of the phage library**

II: **Target biotinylation**

III: **Primers**

IV: **Phage titers**

V: **Peptide cyclisation and mass spectrometric analysis**

VI: **Chemical synthesis of peptides.**

VII: **Expression and purification of JNK3**

VIII: **Fluorescence polarization assay**

IX: **Fluorescence polarization data**

X: **NMR data**

I: Preparation of the phage library

The genes encoding domains D1 and D2 of cysteine-free *pIII* were amplified by PCR using the primers *d1d2fw* and *d1d2rev*. The forward primer *d1d2fw* adds a 3’ Gly-Ser-Gly linker to the amplificate and the reverse primer *d1d2rev* a 5’ *SfiI* restriction site (GGCCTCGGGGGCC). For the PCR reaction the following mixture was prepared: *d1d2fw* (600 nM), *d1d2rev* (600 nM), dNTP mix (250 µM each), 20 ng *fdg3p0ss21* and 2 mL 10x Pfu buffer finally filled up to 20µL with ddH_2O. After addition of Pfu polymerase (2 units) the PCR tube was immediately placed in a PCR thermocycler (initial denaturation of 2 min at 95 °C, 25 cycles of 50 s at 95 °C, 50 s at 58 °C and 2.5 min at 72 °C, and final elongation for 7 min at 72 °C). Subsequently, the PCR product was purified on a 1% agarose gel (TBE buffer). The amplificate running around 700 bp (see Figure S1) was excised and purified with a gel extraction kit (QIAquick Gel Extraction Kit, Qiagen). In a following step, the DNA sequences encoding the randomized peptide libraries were added to the 3’ end of the previously prepared D1-D2 amplificate using the degenerated primers ella2x6. This primer adds at the 5’-end fused to the variable peptide sequences a *SfiI* restriction site (GGCCTCGGG*GGCC*).

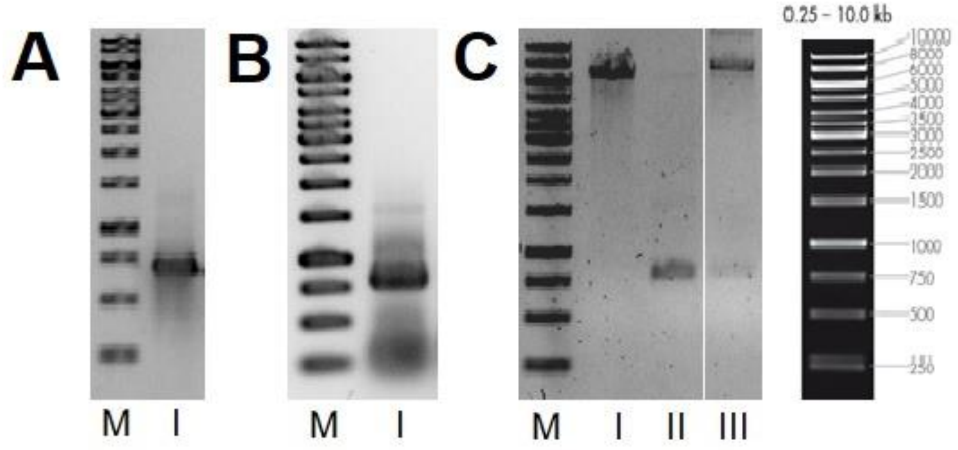

Figure S1: Cloning results. (**A**) I: PCR product of the domain D1 and D2 of cysteine-free *pIII*. (**B**) I: PCR product of the ella2x6 library fused to the PCR amplificate of A(I); (**C**) Ligation of the library inserts and fd phage vector. I: linearized *fd0D12* vector; II: ella2x6 library insert; III: ligation product.
Figure Source: Supporting Information Figure S1 of *ACS Omega*, 2018, 3 (10), 12361–8**,** DOI: 10.1021/acsomega.8b01814 <https://pubs.acs.org/doi/abs/10.1021%2Facsomega.8b01814>. Further requests for permissions related to this material should be directed to the ACS.

For the second PCR reaction, the following mixture was prepared: *ella2x6* (600 nM), *d1d2rev* (600 nM), dNTP mix (250 µM each), 300 ng of the first PCR product and 50 µL 10xTaq buffer finally filled up to 500 mL with ddH_2O and a final DMSO concentration of 5%. After addition of 2 µL Taq polymerase (10 units) the PCR tube was immediately placed in a PCR thermocycler (initial denaturation of 2 min at 95 °C, 30 cycles of 50 s at 95 °C, 50 s at 58°C and 2.5 min at 72 °C, and final elongation for 7 min at 72 °C). The resulting PCR product was purified by electrophoresis on a 1% agarose gel in TBE buffer. The product band appears around 750 bp (see **Fig. S1**). It was excised and purified with a gel extraction kit (QIAquick Gel Extraction Kit, Qiagen). Subsequently the purified PCR product was digested with *SfiI* (New England Biolabs). To 15 µg of the respective purified PCR (ella2x6 library) product we added 20 µL 10x CutSmart buffer (New England Biolabs) and the mixture was diluted to 200 µL. SfiI (10 µL, 20 U/µL) was added, the reaction mixed well by pipetting and it was incubated over night at 50 °C in a thermocycler with a lid heated to 50 °C. The digested PCR product was purified with a DNA purification kit (QIAquick Gel Extraction Kit, Qiagen). The fd-phage fd0D12 vector was amplified as follows: First, 5 mL LB/chloramphenicol preculture was inoculated with a single colony of *Escherichia coli* TG1 bearing the fd0D12 vector. After incubation over-night (37°C, 200 rpm) 500 mL LB/chloramphenicol culture in a 2 L shaking flask was inoculated with the preculture. After incubation for 16 h at 37°C / 200 rpm the phage vector was extracted using a plasmid preparation kit (QIAGEN Plasmid Maxi Kit). The purified fd0D12 vector is first digested with *EcoRI* and in a second step with *SfiI*. To 200 µg of the fd0D12 vector we added 50 µL 10x CutSmart buffer (New England Biolabs) and the mixture was diluted to 500 µL. *EcoRI - HF* (50 mL, 20000U/mL, New England Biolabs) was added, the reaction was mixed well by pipetting and incubated for 2h at 65 °C on a heating block. Subsequently, we added 50 µL 10x CutSmart buffer (New England Biolabs) and the mixture was diluted to 1000 µL. After addition of *SfiI* (100 µL, 2000 U, 20000 U/mL) the reaction is incubated for further 4 h at 50°C. The two *SfiI* sites are not identical. The digested linear vector was purified by electrophoresis on a 1% agarose gel in TBE buffer. The product band appears around 6 kbp (see Figure S1) and was excised and purified with a gel extraction kit (QIAquick Gel Extraction Kit, Qiagen). Ligation of the vector and the respective library insert was performed as follows: 30 µg *SfiI* digested fd0D12 vector was ligated with 9 µg (3-fold molar excess) of the respective library insert. After mixing the insert and vector 60 µL of 10x T4 Ligase Buffer (New England Biolabs) was added and the mixture diluted to 600 µL. T4 ligase (20 µL, 10.000 U) was added, the reaction mixed by pipetting and incubated for 24 h at 16 °C. The T4 ligase was inactivated by incubation at 65 °C for 10

min. The ligated phage vectors were desalted via butanol precipitation. After adding a 10-fold volume of butanol and vortexing for 5 seconds, the mixture was centrifuged at 17.000 g and 4°C for 20 min. Subsequently, the supernatant was discarded and a 5-fold volume of 70%-ethanol/30% ddH_2O was added and the mixture again centrifuged at 17.000 g and 4°C for 20 min. After this step the supernatant was discarded. Residues of ethanol and water were removed under vacuum and the plasmid DNA was dissolved in 500 µL ddH_2O. The purified and desalted DNA was electroporated into freshly prepared *E.coli* TG1 cells. 5 mL of electrocompetent cells were mixed with 500 µL purified DNA and electroporated using 400 µL/cuvette. After each electroporation, cells were resuspended in pre-warmed SOC media and incubated for 1 h at 37 °C with 200 rpm shaking. The size of the library was determined by measuring the total volume of electroporated cells, taking an aliquot of 20 µL, and plating a series of 10-fold dilutions on small 2xTY/chloramphenicol agar plates. In order to assess the quality of the library 50 clones of each library were sequenced. After addition of glycerol (fin. conc.: 20%) the libraries were stored in aliquots of 1 mL at -80°C.

II: Target biotinylation and immobilization on magnetic streptavidin beads

The biotinylation of the target proteins was always performed directly before panning. The target protein (0.5 mg) at a concentration of 10 µM in PBS (pH 7.4) was incubated with 0.8 equivalents of Sulfo-NHS-LC-biotin for 1 h at room temperature. The Sulfo-NHS-LC biotin solution in water was always freshly prepared. To the biotinylated target protein we subsequently added the required amount (0.5 mg) of streptavidin-functionalized magnetic beads for panning. The excess of biotinylated target protein over the binding capacity of the applied amount of beads was in all experiments > 10. The success of the biotinylation was verified by capture on magnetic streptavidin beads. To 1 mg of biotinylated and non-biotinylated protein as negative control we added 25 µL (250 mg) of magnetic streptavidin beads. After 15 minutes of incubation at room temperature and washing 5 times with 1 mL PBS each, the beads of the positive and negative control were analyzed by SDS-PAGE.

III: Primers

The ella2x6 primer codes for the peptide library. This primer was synthesized by randomized trimers (Ella Biotech GmbH, Am Klopferspitz 19, 82152 Martinsried). Variable positions (NNN) may contain the following trimers: AAA (Lys), AAC (Asn), ACT (Thr), ATC (Ile), ATG (Met), CAG (Gln), CAT (His), CCG (Pro), CGT (Arg), CTG (Leu), GAA (Glu), GAC (Asp), GCT (Ala),GGT (Gly), GTT (Val), TAC (Tyr), TCT (Ser), TGG (Trp), TTC (Phe).

d1d2fw:

5'-GGCGGTTCTGGCGCTGAAACTGTTGAAAGTAG-3'

d1d2rev:

5'-GAAGCCATGGCCCCCGAGGCCCCGGACGGAGCATTGACAGG-3'

ella2x6:

5'-TATGCGGCCCAGCCGGCCATGGCAGCGTGT$(NNN)_6$TGC$(NNN)_6$TGTGGCGGTTCTGGCGCTG-3'

seqba:

5'-TAATTGCTCGACCTCCTCTC-3'

IV: Phage titers

Table S1. Determined phage titers after each phage selection round. The input value before each selection was always between 10^9 and 10^{10}.

	**Round 1**	**Round 2**	**Round 3**
c-Jun N-terminal kinase 3 (JNK3)<br>Library: ella2x6	$5.8 \cdot 10^4$	$3.9 \cdot 10^5$	$6.8 \cdot 10^6$

V: Peptide Cyclisation and Mass Spectrometric Analysis

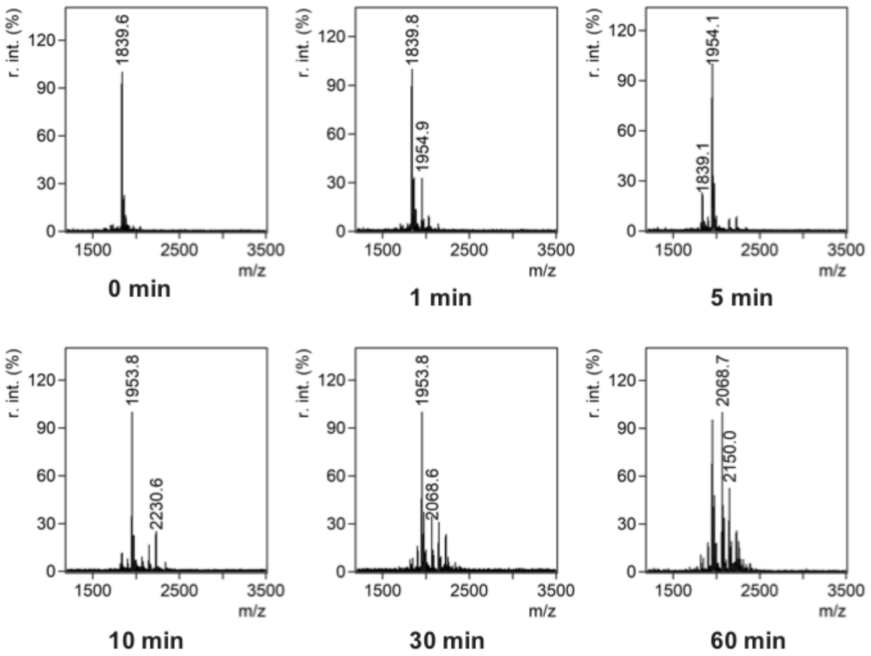

Figure S2. Reaction process of TBMB with TPB measured by MALDI-TOF over a time of 60 min. Reaction conditions: 20 mM NH_4CO_3, 5 mM EDTA, 15% acetonitrile, pH 8.0, 30°C, 0.5 mM TPA, 1.5 equivalents TBMB. Molecular weight TPB-TBMB: 1952.19 Da, TPB (NH_2-ACEGMINSCEKSDYECG-$CONH_2$, 1839.1 Da)

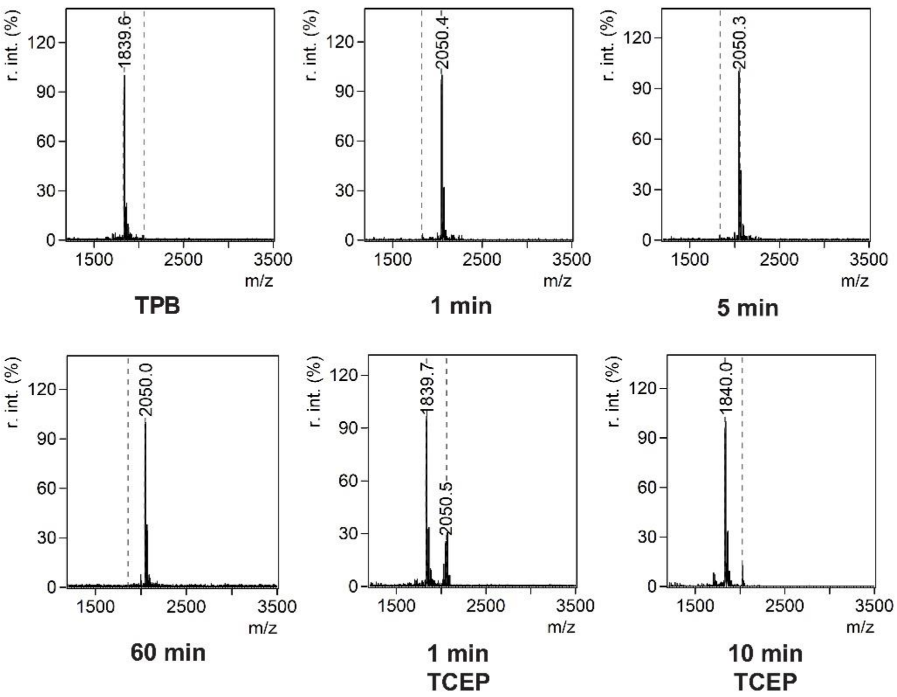

Figure S3. The reversible cyclisation of TPB by TPSMB investigated by MALDI-TOF. Reaction conditions: 20 mM HEPES, 15% acetonitrile, pH 7.0, 30°C, 0.5 mM TPB, 1.5 equivalents TPSMB. Molecular weight TPB-TPSMB: 2049.1 Da, TPB (NH_2-ACEGMINSCEKSDYECG-$CONH_2$, 1839.1 Da). Reduction with TCEP (5 mM) was performed in 20 mM HEPES, 15% acetonitrile, pH 7.0, 30°C.

VI: Chemical Synthesis of Peptides.

Peptides with free N-terminus and amidated C-terminus were synthesized on a 50 µmol scale by standard Fmoc solid-phase chemistry on an automated peptide synthesizer (Syro I, MuliSynTech GmbH, Witten, Germany). Peptides planned to bear N-terminal fluorescein were treated before deprotection and cleavage from resin with 250 mM 5/6-carboxyfluorescein (Sigma-Aldrich) in 5 mL 400 mmol N-methylmorpholine / dimethylformamide for 12 h at room temperature. After deprotection and cleavage from the 4-(2’,4’-dimethoxyphenyl-Fmoc-aminomethyl)-phenoxymethyl (Rink amide) resin (TentaGel™ HL RAM, RAPP Polymere, Tuebingen, Germany) with trifluoroacetic acid (85% v/v, phenol 5.5% w/v, 4.5 % (v/v) triisopropylsilane, 4.5 % (v/v) H_2O), resulting deprotected peptides were precipitated three times in cold diethylether (-20°C). Subsequently, the crude peptides were purified by reversed-phase HPLC (Interchim, PuriFlash 4250) using a Reprosil100 column (C18, 5µm, 20 x 250mm, Dr. Maisch, Ammerbuch, Germany) and a linear gradient elution with a mobile phase composed of eluent A (99.9 % (v/v) H_2O and 0.1 % (v/v) trifluoroacetic acid) and eluent B (80%(v/v)

acetonitrile, 20%(v/v) H_2O and 0.1%(v/v) trifluoroacetic acid) at flow rate of 15 mL/min. The molecular masses were determined by MALDI-TOF mass spectrometry (Bruker Daltonics - autoflex II, Massachusetts, USA). Cyclisation with TPSMB: The peptide (1 mM) dissolved in 3 mL 70% buffer R (20 mM HEPES, pH 7) / 30 % acetonitrile was reacted with 1.1 equivalents TPSMB dissolved in 1 mL acetonitrile under shaking at 30°C. Chemically modified peptides were purified as described above. We intended to synthesize twelve peptides, but unfortunately the selected sequences (JK1, JK2, JK3, JK7, JK10, JK16, JK18, JK20, JK22, JK23, JK25, JK27) turned out to be challenging, in some cases impossible to prepare in decent yields. Very similar byproducts required multiple purification steps. Because of that, the availability of the peptide samples were limited and not all of the selected peptide could be tested for their JNK3 affinity. The purity of the modified and unmodified peptides were assessed by analytical RP-HPLC (Agilent 1100 system, Agilent Technologies, Santa Clara, California, USA), using a C_{18} column and the same buffer system as for preparative RP-HPLC.

VII: Expression and Purification of JNK3

The expression and purification of $JNK3^{339-402}$ was performed as described in literature. [Lange, A.; Günther, M.; Büttner, F. M.; Zimmermann, M. O.; Heidrich, J.; Hennig, S.; Zahn, S.; Schall, C.; Sievers-Engler, A.; Ansideri, F.; et al. Targeting the Gatekeeper MET146 of C-Jun N-Terminal Kinase 3 Induces a Bivalent Halogen/Chalcogen Bond. *Journal of the American Chemical Society* **2015**, *137* (46), 14640–14652.]

VIII: Fluorescence polarization assay

Fluorescence polarization assays were performed in 96-wellplates (black, non-binding, Greiner bio-one). The measurements were performed in a CLARIOstar microplate reader (BMG Labtech, Ortenberg, Germany; excit. filter: 480 nm and an emiss. filter: 530 nm). Purified JNK3 was diluted in buffer (HEPES-Buffer, 25 mM) containing additional 0.1 mg/mL BSA. In the case of direct binding fluorescence polarization experiments, each protein concentration with a maximum concentration between 5 µM and 200 µM and a minimum concentration between 0.08 nM – 0.76 nM was measured in triplicates. The K_D was obtained by curve fit based on eleven protein concentrations. For competitive binding fluorescence polarization experiments, sample concentrations between 200 µM and 10 nM were measured in triplicates. A steady concentration of 10 nM was used for JNK3 and of 5 nM for the fluorescein-labelled compound PIT0105006 (K_D= 3.0 ± 0.2 nM). [Ansideri, F.; Lange, A; El-Gokha

A.; Boeckler F. M.; Koch, P. Fluorescence polarization-based assays for detecting compounds binding to inactive c-Jun N-terminal kinase 3 and p38α mitogen-activated protein kinase. *Anal Biochem.* 2016, 503, 28-40.]Control measurements were performed with PIT0104002 (concentration between 10 µM and 0.5 nM) and confirmed an average K_D of 12 nM. For all experiments DMSO (>99.5% (GC)) was consistently used with a steady concentration of 5% (v/v). Before each measurement, the plates were shaked at 350 rpm for 30 min at room temperature on a plate shaker (Eppendorf MixMate). Before the measurement, the plates were incubated within the microplate reader for 2 min at 25°C and the focal height was adjusted to yield the optimal fluorescence intensity. Measured data was plotted and K_D values were obtained by curve fitting of a standard four parameter logistic nonlinear regression model using the program Origin 9.1.

IX: Fluorescence polarization data

Table S2: Overview of the results obtained in direct titration fluorescence polarization assays. SE = standard error from curve fit, SD = standard deviation from experiments 1-3.

	**K_D [µM]**							
	**Exp. 1**	**SE**	**Exp. 2**	**SE**	**Exp. 3**	**SE**	**Mean K_D [µM]**	**SD**
**JK1**	**1.7**	0.6	**0.7**	0.078	**2.6**	0.1	**1.7**	**0.78**
**JK16**	**6.7**	4.1					**6.7**	
**JK18**	**4.1**	1.7					**4.1**	

Table S3: Overview of the results obtained in competitive fluorescence polarization assays. [a] Conversion of obtained IC_{50} mean values to K_I were performed using a web-based application available at: http://sw16.im.med.umich.edu/software/calc_ki/ (19.07.2018) [Cer, R. Z., Mudunuri, U., Stephens, R., & Lebeda, F. J. (2009). IC_{50}-to-K_i: a web-based tool for converting IC_{50} to K_i values for inhibitors of enzyme activity and ligand binding. Nucleic Acids Research, 37(Web Server issue), W441–W445], SE = standard error from curve fit.

	**IC_{50} [µM]**					
	**Exp. 1**	**SE**	**Exp. 2**	**SE**	**Mean IC_{50} [µM]**	**K_I [µM][a]**
**JK1**	**15.6**	2.6	**41.3**	4.1	28.45	**6.6**
**JK10**	**21.8**	4.2	**25.5**	6.1	23.65	**5.5**
**JK20**	**27.8**	5.4			27.8	**6.5**

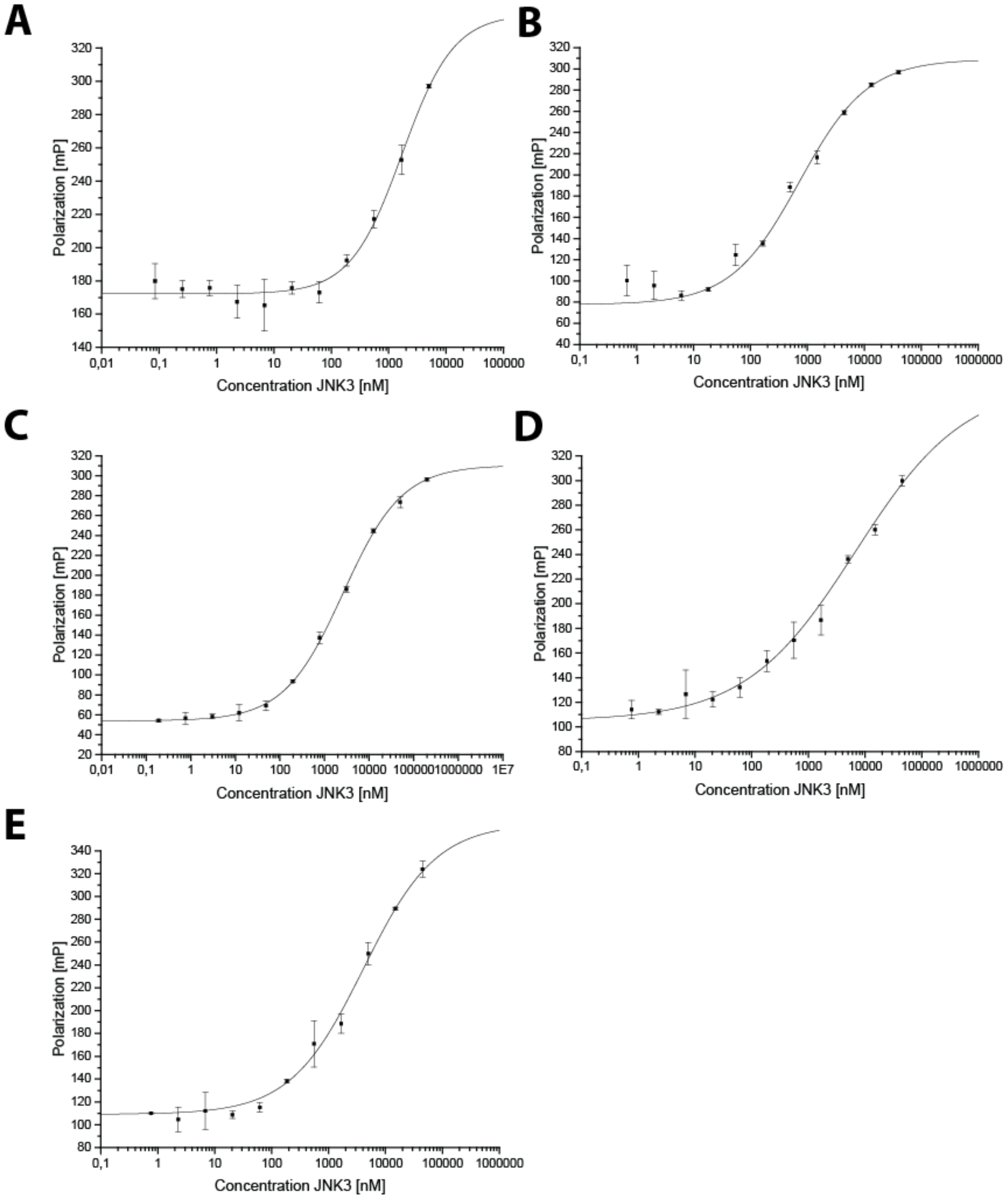

Figure S4. Overview of the results obtained in direct titration fluorescence polarization assays. (**A**) JK1F-TPSMB K_D = 1.7 µM (SE: 0.6 µM); (**B**) JK1F-TPSMB K_D = 720 nM (SE: 78 nM); (**C**) JK1F-TPSMB K_D = 2.6 µM (SE: 0.1 µM); (**D**) JK16F-TPSMB K_D = 6.7 µM (SE: 4.1 µM); (**E**) JK18F-TPSMB K_D = 4.1 µM (SE: 1.7 µM); SE = standard error from curve fit.

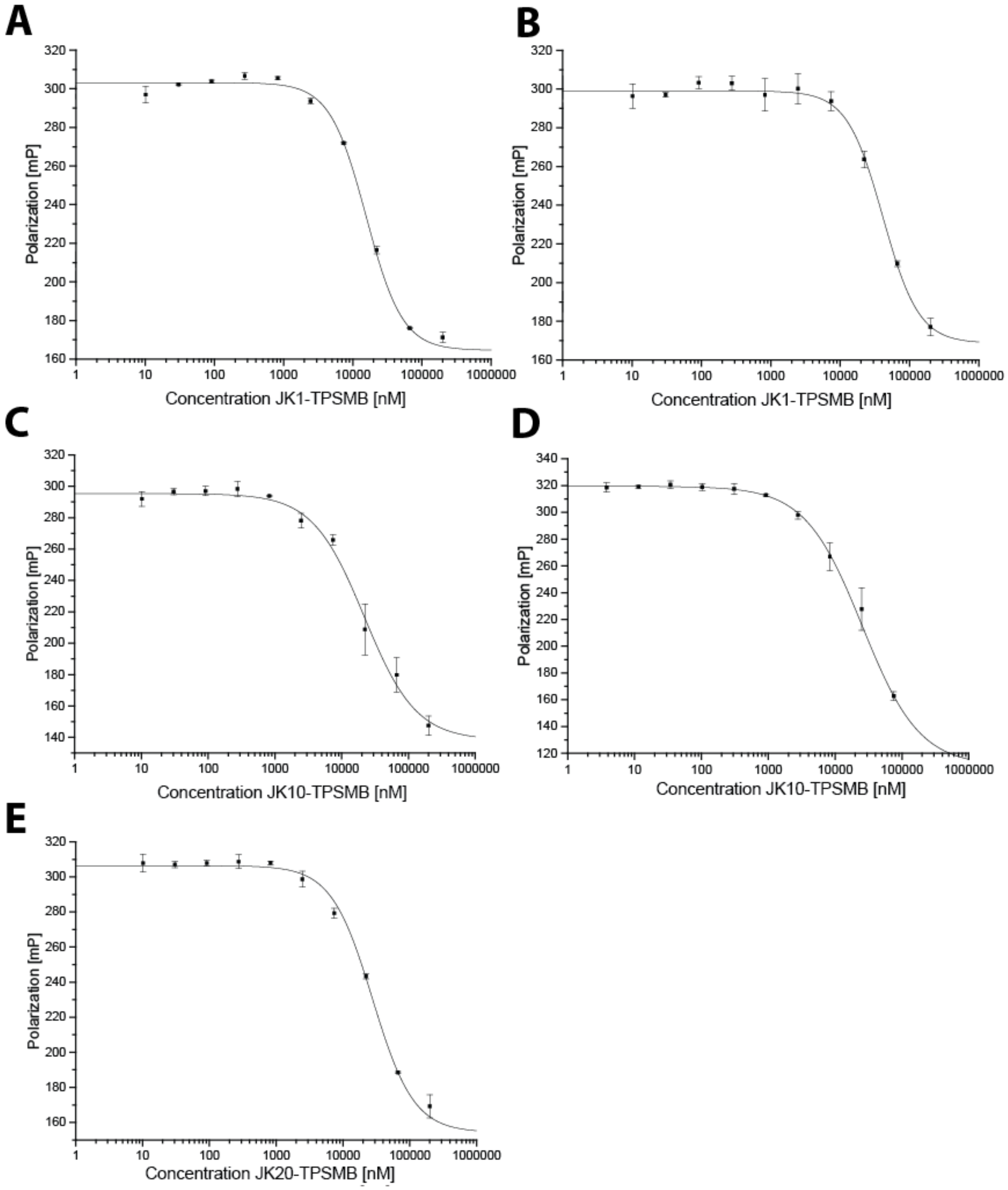

Figure S5. Overview of the results obtained in competitive fluorescence polarization assays. (**A**) JK1-TPSMB IC_{50} = 15.6 µM (SE: 2.6 µM); (**B**) JK1-TPSMB IC_{50} = 41.3 µM (SE: 4.1 µM); (**C**) JK10-TPSMB IC_{50} = 21.8 µM (SE: 4.2 µM); (**D**) JK10-TPSMB IC_{50} = 25.5 µM (SE: 6.1 µM); (**E**) JK20-TPSMB IC_{50} = 27.8 µM (SE: 5.4 µM), SE = standard error from curve fit.

X: NMR data

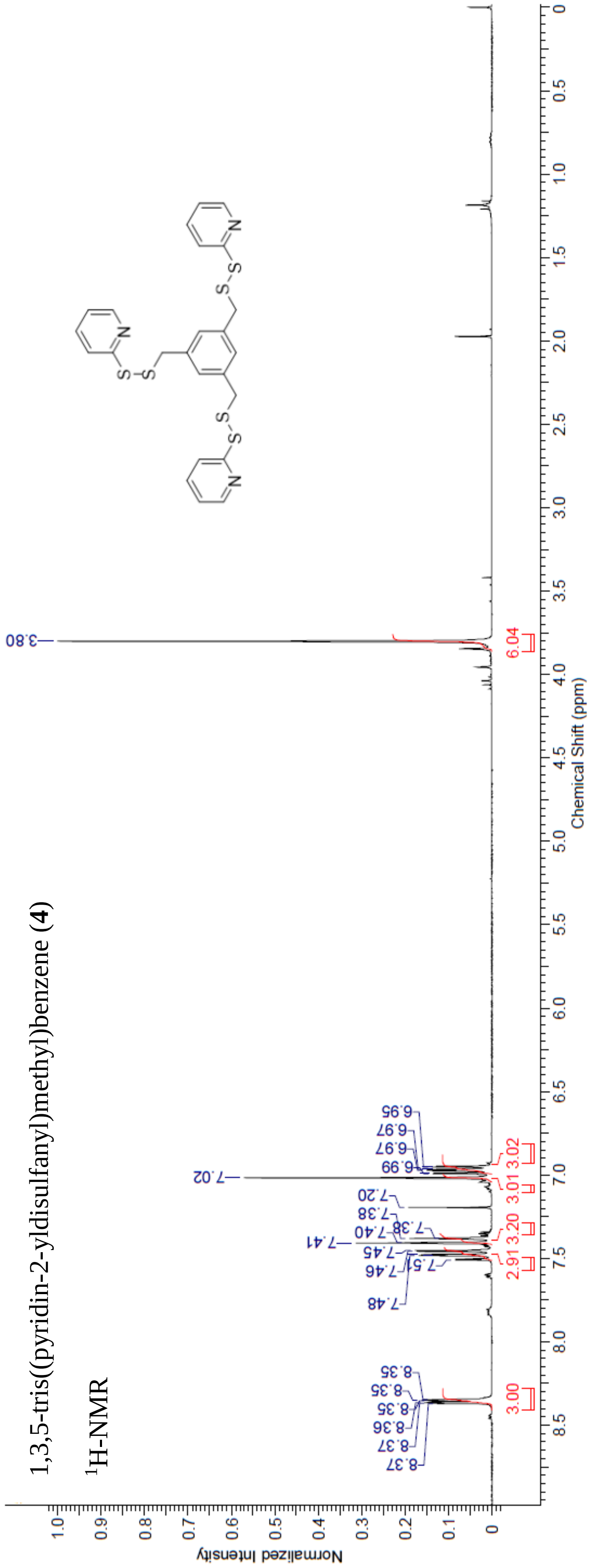
1,3,5-tris((pyridin-2-yldisulfanyl)methyl)benzene (4)
^{1}H-NMR
Chemical Shift (ppm)
Normalized Intensity
3.80
7.02
7.41
6.04
3.00
2.91
3.20
3.01
3.02

1,3,5-tris((pyridin-2-yldisulfanyl)methyl)benzene (**4**)

^{13}C-NMR

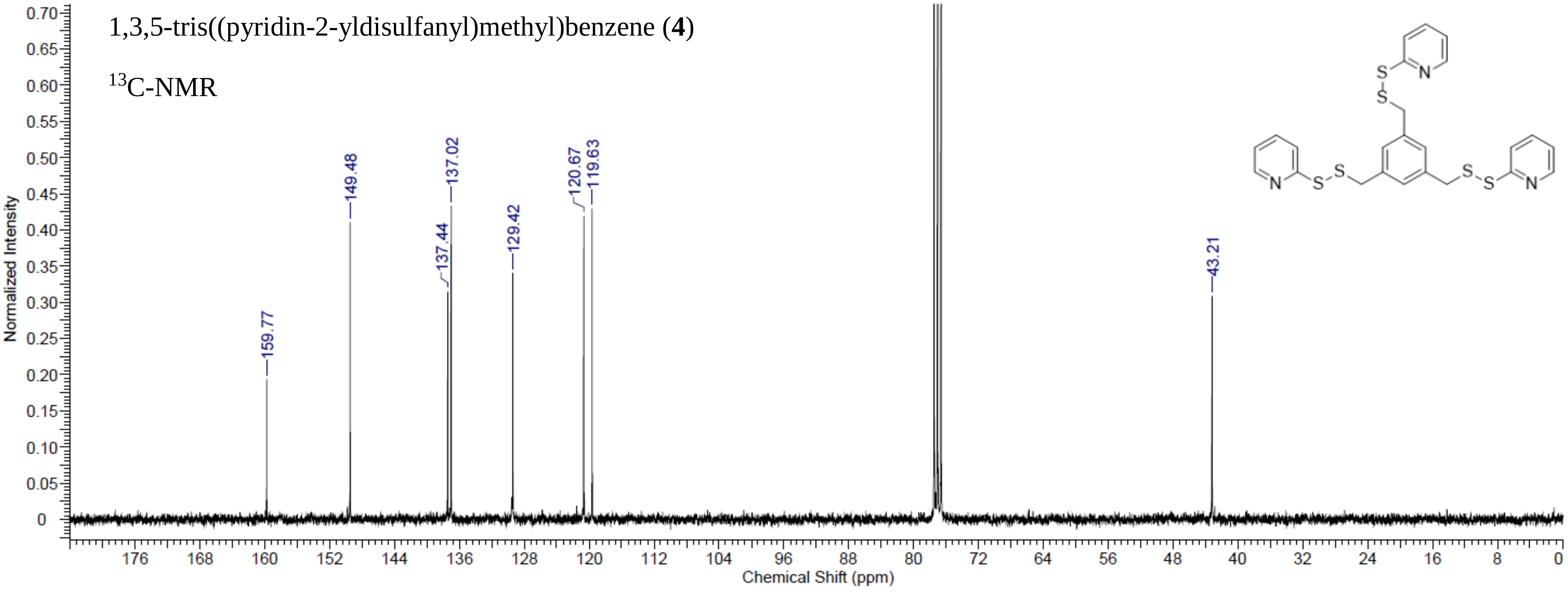

Supplement: Supplementary file 1 [file Data_Sheet_1.pdf]
